# Insulin Receptor Tyrosine Kinase Substrate Enhances Low Levels of MDM2-Mediated p53 Ubiquitination

**DOI:** 10.1371/journal.pone.0023571

**Published:** 2011-08-24

**Authors:** Ke-Sheng Wang, Gang Chen, Hai-Lian Shen, Ting-Ting Li, Fei Chen, Qin-Wan Wang, Zhi-Qin Wang, Ze-Guang Han, Xin Zhang

**Affiliations:** 1 Shanghai-MOST Key Laboratory for Disease and Health Genomics, Chinese National Human Genome Center at Shanghai, Shanghai, China; 2 School of Bioengineering, East China University of Science and Technology, Shanghai, China; 3 Rui-Jin Hospital, Shanghai Jiaotong University School of Medicine, Shanghai, China; University of Minnesota, United States of America

## Abstract

The tumor suppressor p53 controls multiple cellular functions including DNA repair, cell cycle arrest and apoptosis. MDM2-mediated p53 ubiquitination affects both degradation and cytoplasmic localization of p53. Several cofactors are known to modulate MDM2-mediated p53 ubiquitination and proteasomal degradation. Here we show that IRTKS, a novel IRSp53-like protein inhibited p53-induced apoptosis and depressed its transcription activity. IRTKS bound directly to p53 and increased p53 ubiquitination and cytoplasmic localization. Further studies revealed that IRTKS interacted with MDM2 and promoted low levels of MDM2-mediated p53 ubiquitination in vitro and in vivo. In unstressed cells with low levels of MDM2, IRTKS was found to stabilize the interaction of p53 and MDM2. In stressed cells, IRTKS dissociated from p53, and high levels of MDM2 induced by p53 activation mediate IRTKS poly-ubiquitination and subsequent proteasomal degradation. These data suggest that IRTKS is a novel regulator of p53, modulating low level of MDM2-mediated p53 ubiquitination in unstressed cells.

## Introduction

The activity of p53 is controlled by a complicated network during normal homeostasis and stress response [1]. Multiple post-translational modifications of p53, including phosphorylation, acetylation, methylation, glycosylation, ubiquitination and ubiquitin-like modification, have been documented for p53 regulation [2], [3]. Among these modifications, ubiquitination plays a pivotal role in controlling p53 activity under different conditions [4].

Ubiquitin conjugates to proteins either in a monomeric manner (monoubiquitination) to cause conformational change to the substrate or in a polyubiquitin chain which signals the substrate to be degraded by the 26S proteasome. While polyubiquitination serves as a signal for proteasomal degradation which requires an at least four-subunit-long multiubiquitin chain per individual lysine residue, monoubiquitination has been shown to be involved in a variety of cellular processes, including protein trafficking, DNA repair and transcriptional regulation [5], [6]. MDM2, a key regulator of p53, catalyzes both monoubiquitination and polyubiquitination on p53 in a dose-dependent manner. High levels of MDM2 mediate p53 polyubiquitination and proteasomal degradation, whereas low levels of MDM2 induce the monoubiquitination and nuclear export of p53 [7]. Some cofactors, including L5, L11, L23, p300/CBP, Yin Yang 1, MDMx, UBE4B, have been characterized to regulate MDM2-mediated ubiquitination and degradation [8], [9]. But the regulation of low levels of MDM2-mediated p53 monoubiquitination and cytoplasmic translocation has been rarely reported.

IRSp53-like proteins contain a conserved IRSp53/MIM homology domain (IMD) at the N-terminus, and a canonical SH3 domain near the C-terminus [10]. The IMD belongs to the larger family of Bin-amphyipysin-Rvs67 (BAR) domains that can bundle actin filaments and induce membrane protrusions [11]. The IRSp53 family is involved in the formation of filopodia and lamellipodia [12]. Insulin receptor tyrosine kinase substrate (IRTKS), an IRSp53-like protein, has been recognized as a substrate for the insulin receptor tyrosine kinase and its overexpression induces clustering of short actin bundles [13]. However, IRTKS is largely localized in the nucleus wherein its function remains unknown.

In this study, we first found that IRTKS suppressed the p53 induced apoptosis and transactivation activity. IRTKS interacted directly with both p53 and MDM2 through its IMD domain, and enhances low level MDM2-mediated p53 ubiquitination in vivo and in vitro. Interestingly, the effect of IRTKS on the p53-MDM2 axis is dependent on the protein levels of MDM2. At normal physiological status, the interaction between p53 and low level MDM2 was enhanced by IRTKS. During stress, p53 dissociated with IRTKS. And high levels of MDM2 induce ubiquitin-mediated degradation of IRTKS. These data together suggested that the scaffold protein, IRTKS may balance the MDM2 activity in mediating p53 ubiqitination.

## Results

### IRTKS suppressed p53-induced apoptosis and negatively regulated the transcription activity of p53

**Figure 1:**
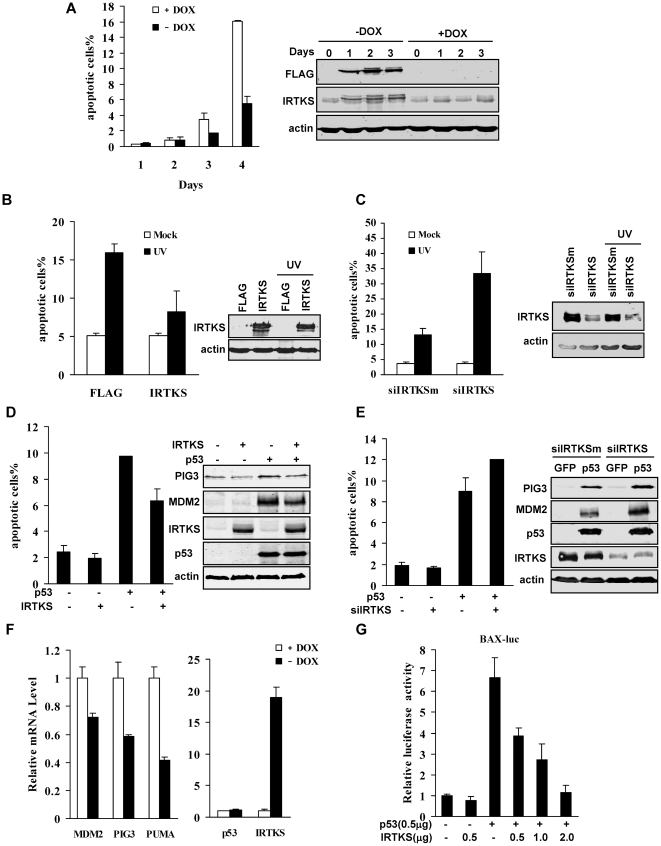
IRTKS suppressed p53-induced apoptosis and transactivation activity. (A) IRTKS inhibited cell death triggered by serum starvation. The IRTKS Tet-off HT1080 cells were cultured in serum-free medium in the presence or absence of doxycycline (DOX, 10 ng/ml). The cells were collected and analysed by FACS after staining with propidium iodide (PI) at the indicated time. The sub-G1 population was counted as the percentage of cell death. The inducible expression of flag-tagged IRTKS was assessed by Western blotting with anti-FLAG and anti-IRTKS antibodies (right panel). (B, C) IRTKS inhibited UV-induced apoptosis. HT1080 cells with overexpression (B) or knockdown (C) of IRTKS were exposed to UV irradiation and analyzed for apoptosis by Annexin V-FITC/PI (propidium iodide) staining. Representative results of four independent experiments were shown. P<0.005. The expression of IRTKS was assessed by Western blotting (right panel). (D) Overexpression of IRTKS inhibited p53-induced apoptosis. SAOS-2 cells infected with adenoviruses for IRTKS or/and p53 overexpression were analyzed with PI staining by FACS. Data were shown as mean ± s.d. (n = 3), p<0.01. The expression of p53, IRTKS and p53 inducible gene products (PIG3 and MDM2) were analyzed by Western blotting (right panel). (E) Knockdown of IRTKS enhanced p53-induced apoptosis. SAOS-2 cells were treated with IRTKS-siRNA for 24 h, then infected with Ad-p53 or control virus Ad-GFP, and analyzed for apoptosis. Data were shown as mean ± s.d. (n = 3), p<0.01. The expression of p53, IRTKS and p53 inducible gene products (PIG3 and MDM2) were analyzed by Western blotting (right panel). (F) IRTKS inhibited the expression of p53-inducible genes. Total RNA from the IRTKS Tet-off HT1080 cells in the presence (10 ng/ml) or absence of doxycycline was subjected to real-time quantitative PCR (qPCR) analysis. Data were expressed as mean ± s.d. (n = 3). p<0.01. (G) IRTKS decreased the transactivation of p53. p53 transactivation activity on the BAX promoter was measured by luciferase reporter gene assay in SAOS-2 cells.

To investigate the function of IRTKS, we established a stable HT1080 cell line (HT1080-IRTKS) with expressing ectopic FLAG-tagged IRTKS upon withdrawal of doxycycline [14]. Interestingly, overexpression of IRTKS remarkably suppressed serum depletion-induced cell death (Figure 1A), implying that IRTKS may possess anti-apoptotic activity. HT1080 cells express wild type p53, which mediates the apoptotic response to DNA damage [15]. We found that IRTKS overexpression reduced the number of apoptotic cells upon UV exposure (Figure 1B and Figure S1A). By contrast, IRTKS depletion by RNA interference (RNAi) enhanced UV irradiation-induced apoptosis (Figure 1C and Figure S1B) and the apoptosis-related cleavage of poly(ADP-ribose) polymerase (PARP) (Figure S1C). We also infected p53-null SOAS-2 cells with adenoviruses expressing p53 and IRTKS, and found that IRTKS overexpression inhibited apoptosis mediated by ectopic p53(Figure 1D and Figure S1D) while IRTKS knockdown increased p53-induced apoptosis in SOAS-2 cells (Figure 1E and Figure S1E). These observations demonstrated that IRTKS negatively modulated p53-induced apoptosis.

p53-induced apoptosis is largely dependent on its transactivation of such proapoptotic genes as *BAX*, *PUMA* and *PIG3* [16]–[18]. We sought to determine the effect of IRTKS induction on the expression of p53 target genes. We found that IRTKS overexpression slightly decreased the expression of *PIG3* and *MDM2* in SOAS-2 cells (Figure 1D, right panel). By contrast, deletion of *IRTKS* increased the expression of *PIG3* and *MDM2* in SOAS-2 cells (Figure 1E, right panel). Induction of IRTKS overexpression in HT1080 cells upon doxycycline withdrawal also reduced the levels of *PIG3*, *PUMA* and *MDM2* with no apparent effect on the expression of p53 (Figure 1F). Furthermore, in SOAS-2 cells, using a luciferase reporter system under the control of the *BAX* promoter, we found that IRTKS overexpression dose-dependently inhibited p53-induced luciferase activity (Figure 1G). These data showed that IRTKS attenuated the transactivation of p53 on its downstream genes, suggesting that IRTKS may exert its anti-apoptotic effect through modulating p53 transcriptional activity.

### IRTKS interacted directly with p53 in nucleus

**Figure 2:**
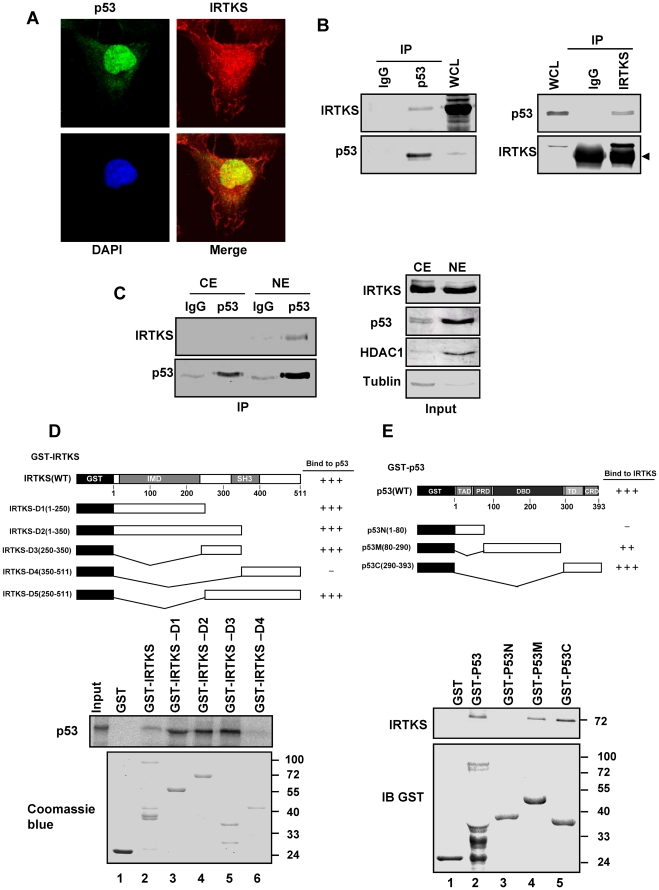
IRTKS directly interacted with p53. (A) Co-localization of IRTKS and p53. HT1080 cells were immunostained with anti-p53 antibody and anti-IRTKS antibody followed by Alexa-488 conjugated anti-mouse antibody and Alexa-546 conjugated anti-rabbit antibody. Images were taken through a confocal microscope. (B) Association of endogenous ITRKS and endogenous p53 in HEK 293 cells. Whole cell lysates (WCL) and immunoprecipitations (IP) with control IgG, p53 antibody, preimmune serum or IRTKS antibody were analyzed by Western blotting. The arrowhead indicates IgG heavy chain. (C) IRTKS interacted with p53 in nucleus. Immunoprecipitations of the nuclear and cytoplasmic extracts from HT1080 cells with p53 antibody were analyzed with IRTKS antibody by Western blotting. (D) Direct interaction between p53 and IRTKS revealed by GST-pulldown assays. Full-length or truncated GST-IRTKS proteins were incubated with in vitro translated ^35^S-labelled p53 in NETN buffer, and then precipitated with glutathione-Sepharose beads. Bound proteins were resolved by 10% SDS-PAGE followed by autoradiography. GST-IRTKS proteins were visualized by Coomassie blue staining. (E) Interaction site mapping on p53. GST-pulldown assays were performed with recombinant His-IRTKS and full-length or truncated GST-p53. The pulldown samples were subjected to Western blotting with anti-IRTKS and anti-GST antibodies.

We were interested in determining whether IRTKS exerted its effects on p53 through its physical association with the protein. We defined the subcellular localization of both proteins by immunofluorescence assays, which showed that both IRTKS and p53 were co-localized in the nucleus (Figure 2A), suggesting that IRTKS could physically associate with p53. Reciprocal co-immunoprecipitations (Co-IPs) using antibodies against IRTKS and p53 confirmed their association in HEK 293 and HT1080cells (Figure 2B, Figure S2A). Co-IPs with nuclear and cytoplasmic extracts from HT1080 cells further revealed that, consistent with the data from immunofluorescence assays (Figure 2A), endogenous IRTKS interacted with p53 exclusively in the nucleus (Figure 2C). Direct interaction of IRTKS with p53 was further demonstrated *in vitro* by GST-pulldown assays (Figure 2D). Additionally, two regions of IRTKS (N-terminal 1–250 and 250–350 amino acid residues), which contain the IMD domain and an uncharacterized domain, respectively, were found to interact with p53 by GST-pulldown experiments with a series of deletion variants of IRTKS (Figure 2D). Consistently, two regions of p53 were involved in the direct interaction of IRTKS and p53. While the p53 deletion variants containing its C-terminal regulatory domain (CRD) strongly bound to IRTKS, the DNA binding domain (DBD) of p53 also interacted with IRTKS (Figure 2E and Figure S2B). These findings indicated that IRTKS physically associated with p53 in the nucleus via their functional domains.

### IRTKS increases the ubiquitination and the cytoplasmic localization of p53

**Figure 3:**
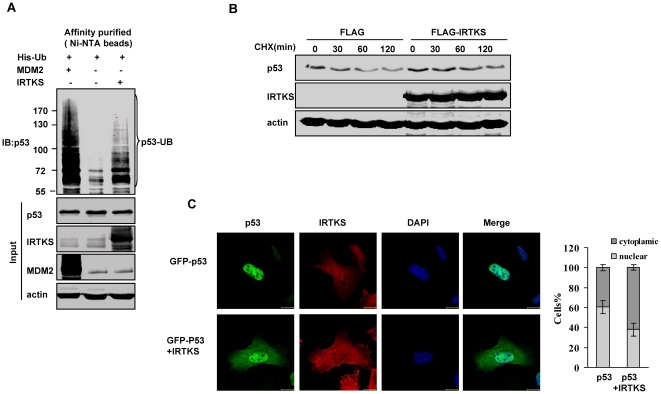
IRTKS enhanced the ubiquitination and cytoplasmic localization of p53. (A) In vivo ubiquitination assay revealed IRTKS-induced p53 ubiquitination. U2OS cells were co-transfected with a 6XHis-Ub expression vector and IRTKS (lane 3). The cell lysates were loaded on nickel (Ni+)-NTA columns. p53 ubiquitination was analyzed with Western blotting by using anti-p53 antibody (DO-1). MDM2 was used as a positive control for p53 ubiquitination (lane1). Note that the IRTKS-induced p53 ubiquitination was in lower molecular weights (<130 kD). (B) IRTKS overexpression resulted in a moderate stabilization of endogenous p53 protein. Half-life of p53 protein was measured in cycloheximide (CHX) treated U2OS cells transfected with FLAG-IRTKS and control plasmid. Protein extracts, prepared at indicated time points after CHX addition, were analyzed by Western blotting. (C) IRTKS increased the cytoplasmic localization of p53. U2OS cells transfected with GFP-p53 and FLAG-IRTKS were stained for IRTKS. The nuclei were counterstained with DAPI and images were taken through a confocal microscope. One hundred cells were counted for each treatment in two separate experiments. The cells with higher levels of GFP-p53 in the cytoplasm versus the nucleus (dark gray bars) and higher levels of p53 in the nucleus versus the cytoplasm (gray bars) were scored.

p53 can be negatively regulated at the transcriptional, translational and post-translational levels [1]. As IRTKS did not alter the abundance of p53 mRNA and protein levels (Figure 1F and Figure S3), we proposed that IRTKS negatively modulated p53 activity at the post-transcriptional level. We found that IRTKS overexpression failed to induce changes in phosphorylation and acetylation of certain amino acid residues of p53 under stress or unstress conditions, including serine 15, 20, 37, 46, 392, and lysine 382 (Figure S3). However, p53 ubiquitination with lower molecular weights (<130 Kd) was significantly increased in HT1080 cells overexpressing IRTKS (Figure S4A). By contrast, IRTKS knockdown reduced the ubiquitination of endogenous p53 (Figure S4B). In vivo ubiquitination assay in U2OS cells further confirmed that, distinct from the p53 ubiquitination pattern by ectopic MDM2, IRTKS indeed mediated p53 ubiquitination with increasing lower molecular weight modifications (Figure 3A).

Previous reports have demonstrated that p53 polyubiquitination is associated with its proteasomal degradation while p53 monoubiquitination induces its nuclear export. Our data here showed that IRTKS did not affect the protein levels of endogenous and ectopic p53 (Figure S3 and S4; Figure 1D and 1E). On the contrary, IRTKS overexpression slightly stabilized endogenous p53 in U2OS cells treated with cycloheximide (Figure 3B). Our immunofluorescence assay revealed that IRTKS overexpression increased the cytoplasmic localization of p53 (Figure 3C). These data indicated that IRTKS-induced p53 ubiquitination affects the subcellular localization of p53.

### IRTKS interacts with MDM2

**Figure 4:**
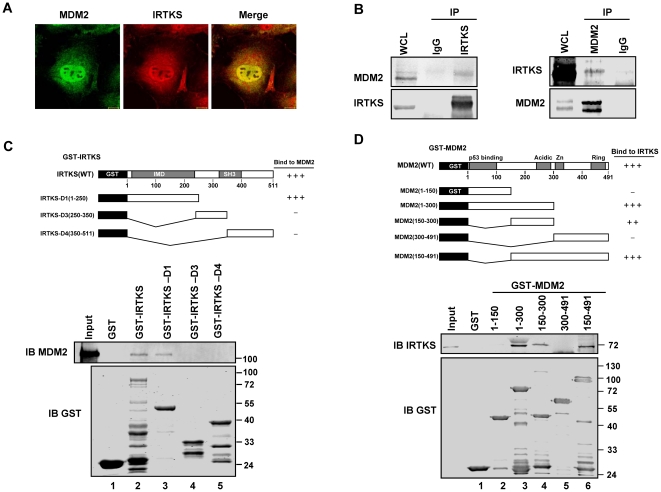
IRTKS interacted with MDM2. (A) Colocalization of IRTKS and MDM2. HT1080 cells were transfected with MDM2 and immunostained with anti-MDM2 antibody and anti-IRTKS antibody. (B) Interaction of endogenous IRTKS and endogenous MDM2 in HT1080 cells. Whole cell lysate (WCL) and immunoprecipitations (IP) with preimmune serum or IRTKS antibody, control IgG, MDM2 antibody were analyzed by Western blotting. (**C**) Mapping of IRTKS domain for MDM2 binding. GST-pulldown assay was performed with recombinant GST-IRTKS variations and His-MDM2 to reveal the direct interaction between IRTKS and MDM2, and map the interaction site of IRTKS. (D) Interaction site mapping on MDM2 for IRTKS.

IRTKS was previously considered as a scaffold protein without enzymatic activity [13], [19]. IRTKS alone also failed to demonstrate any catalytic activity on p53 ubiquitination in our in vitro ubiquitination assays (Figure S6B, lane 1–4). Therefore, IRTKS might function as an adaptor for the ubiquitin E3 ligases to mediate p53 ubiquitination. As known, MDM2 is essential for regulating p53 function and catalyzes p53 ubiqitination for degradation and nuclear export dose-dependently [7], [20], [21]. In MDM2 null cells, IRTKS only increased p53 ubiquitination when MDM2 was co-transfected to the cells (Figure S5), implying that IRTKS affected MDM2-mediated p53 ubiquitination (Figure S5). To assess the possibility that IRTKS could serve as an adaptor of MDM2, we performed MDM2 and IRTKS co-localization assays, which disclosed an almost identical subcellular localization of IRTKS and MDM2, especially in the nucleus (Figure 4A). Co-IP analysis additionally revealed that endogenous IRTKS and MDM2 were reciprocally immunoprecipitated (Figure 4B), suggesting that IRTKS indeed physically associated with MDM2. In vitro GST-pulldown assays with purified recombinant proteins confirmed their physical interaction, where both the IMD domain of IRTKS and 150–300 residues of MDM2 were required for their interaction (Figure 4C and 4D). These data implied that IRTKS could recruit both MDM2 and its substrate p53 through the IMD domain and serve as a cofactor of MDM2.

### IRTKS promoted p53 ubiquitination induced by low levels of MDM2

**Figure 5:**
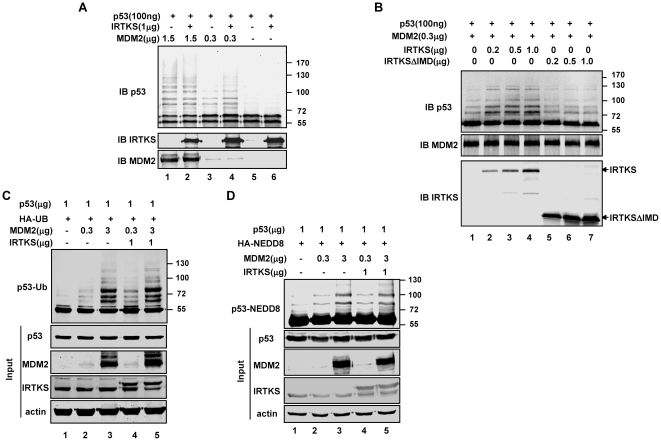
IRTKS promoted MDM2-mediated p53 ubiqutination. (A) IRTKS only enhanced p53 ubiquitination mediated by low levels of MDM2. In vitro ubiquitination assays were performed with recombinant His-p53, His-IRTKS and varying amounts of His-MDM2, and analyzed with p53-specific monoclonal antibody (DO-1) by Western blotting. (B) IMD was essential for IRTKS to promote MDM2-mediated p53 ubiquitination. In vitro ubiquitination assays were performed with p53, low levels of MDM2 and varying amounts of IRTKS (lane 1–3) or IMD deletion mutant IRTKS (lane 4–6). (C) IRTKS promoted low levels of MDM2-mediated ubiquitination of p53 in vivo. H1299 cells were cotransfected with the plasmids for expression HA-ubiquitin, p53, varying amounts of MDM2 and IRTKS or control plasmid. Cells were treated with 25 µM MG-132 for 4 h and the lysates were immunoprecipitated with anti-HA antibody. p53 ubiquitination was analyzed by western blot with anti-p53 antibody(DO-1). (D) IRTKS could not affect MDM2-induced p53 neddylation. H1299 cells were cotransfected with the indicated plasmids and treated with 25 µM MG-132 for 4 h. The cell lysates were immunoprecipitated with anti-HA antibody. p53 neddylation was analyzed by western blot with anti-p53 antibody(DO-1).

We further explored the role of IRTKS in MDM2-mediated p53 ubiquitination by assessing the activity of MDM2 for p53 ubiquitination using in vitro ubiquitination assays with recombinant MDM2 and p53. Consistent with the previous study [7], MDM2 induced p53 monoubiquitination and polyubiquitination dose-dependently (Figure S6A). Using this ubiquitination system, we found that IRTKS only enhanced low molecular weight of p53 ubiquitination (<130 Kd) mediated by low levels of MDM2, but had no effect on p53 ubiquitination induced by high levels of MDM2 (Figure 5A). Moreover, IRTKS variant with IMD deletion failed to enhance MDM2-induced p53 ubiquitination (Figure 5B), indicating that the IMD region which was required for the physical association of IRTKS with both MDM2 and p53, was crucial for IRTKS-induced p53 ubiquitination.

In vivo ubiquitination assays using H1299 cells also revealed that IRTKS promoted p53 ubiquitination only with low levels of MDM2 (Figure 5C, lane 4 compared with lane 2). It has been reported that MDM2 mediates both ubiquitination and neddylation of p53. MDM2-mediated NEDD8 modification inhibits p53 transcription activity [22]. Herein, in vivo neddylation assay showed that IRTKS overexpression could not alter MDM2-mediated neddylation of p53 (Figure 5D). Therefore, as a cofactor of MDM2, IRTKS only enhanced p53 ubiquitination induced by low level MDM2.

### IRTKS enhanced the p53-MDM2 interaction at low levels of MDM2

**Figure 6:**
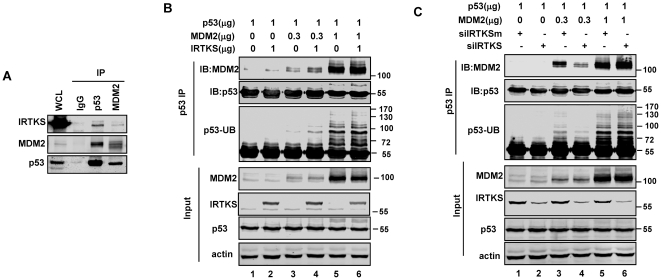
IRTKS enhanced the interaction between p53 and MDM2. (A) Identification of p53, MDM2 and IRTKS within the same complex by co-immunoprecipitation assays with antibodies for p53 and MDM2. (B) Ectopic IRTKS enhanced p53-MDM2 interaction and the p53 ubiquitination in H1299 cells with low levels of MDM2. H1299 cells were co-transfected with the plasmids for expressing p53, varying amounts of MDM2 and IRTKS or control plasmid. Immunoprecipitations with anti-p53 antibody (DO-1) were analyzed by Western blotting with p53 antibody (FL393) and MDM2. The long time exposal of the Western blotting with anti-p53 antibody indicated p53 ubiquitination (the third panel). (C) Knockdown of IRTKS reduced the p53-MDM2 interaction and p53 ubiquitination in H1299 cells at low levels of MDM2. After pre-transfected with IRTKS siRNA or control siRNA for 24 h, H1299 cells were transfected with the plasmids for expressing p53 and varying amounts of MDM2. The p53-MDM2 interactions and p53 ubiquitination were analyzed.

Previous studies showed that the N terminal regions of both MDM2 and p53 provide the primary site for their physical interactions [23], which are distinct from the domain that mediated their binding to IRTKS shown here. Our data indicated that the IMD domain was required for IRTKS binding to p53 and MDM2 and for its effect on MDM2-mediated p53 ubiquitination. The crystal structure of IRTKS indicates that IMD could self-associate into an 180 Å-long zeppelin-shaped dimer [11], [24]. Moreover, an uncharacterized domain of the middle region of IRTKS could provide a secondary site for interaction with p53. These observations raised the intriguing possibility that IRTKS could affect the p53-MDM2 interaction in vivo. Co-IP assays revealed that endogenous p53, MDM2 and IRTKS were all immunoprecipitated with antibodies against p53 or MDM2 (Figure 6A), suggesting that they were present in the same protein complex. We then evaluated the effect of IRTKS on the interaction between MDM2 and p53. Interestingly, a cotransfection assay in H1299 cells showed that the interaction between MDM2 and p53 was significantly enhanced by ectopic IRTKS with low levels of MDM2 (Figure 6B, lane 4 compared with lane 3), whereas no effect on the association of high levels of MDM2 with p53 was detected (Figure 6B, lanes 5 and 6). Additionally, consistent with our findings in in vitro and in vivo ubiquitination assays, p53 ubiquitination at low molecular (<130 kD) was increased at low levels of MDM2 with IRTKS overexpression (Figure 6B, lane 4 compared with lane 3). In contrast, IRTKS knockdown attenuated the p53-MDM2 interaction and p53 ubiquitination at low levels, not high levels, of MDM2 (Figure 6C). These data together indicated that IRTKS induced p53 ubiquitination through enhancing the interaction between p53 and low levels of MDM2 in unstressed cells.

### MDM2 is an E3 ubiquitin ligase for IRTKS degradation

**Figure 7:**
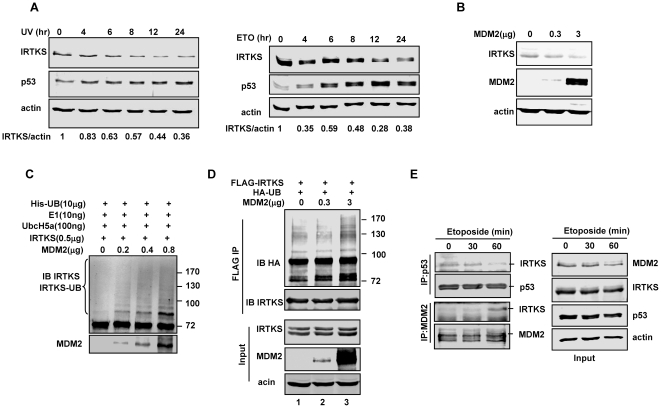
MDM2 induced polyubiquitination and degradation of IRTKS. (A) Downregulation of IRTKS by DNA damage in HT1080 cells. Cells were treated with UV-irradiation (UV, 60 J/M^2^) or etoposide (ETO, 30 µM), collected at indicated time and analysed by Western blotting. (B) Ectopic MDM2 decreased endogenous IRTKS expression. The expression of endogenous IRTKS in HT1080 cells with MDM2 overexpression were analysed by Western blotting. (C) MDM2 directly mediated IRTKS ubiquitination. In vitro ubiquitination assays were performed with recombinant His-IRTKS and varying amounts of His-MDM2, and analyzed with anti-IRTKS antibody by Western blotting. (D) MDM2 induced IRTKS ubiquitination in vivo. In vivo ubiquitination assay was performed with co-transfection of HA-Ubiquitin, FLAG-IRTKS and varying amounts of MDM2 in H1299 cells. The cells were treated with 25 µM MG132 for 4 h before lysis. FLAG-IRTKS was immunoprecipitated with FLAG-beads and ubiquitination of IRTKS was analyzed by western blotting with HA antibody. (E) DNA damage dissociated IRTKS from p53 and enhanced the IRTKS-MDM2 interaction. U2OS cells were treated with 25 µM MG-132 for 4 h and subsequently with 30 µM etoposide as indicated. The p53-IRTKS and MDM2-IRTKS interactions were analyzed.

The protein levels of IRTKS were significantly decreased after DNA damage in HT1080 cells (Figure S1B and Figure 7A) but not in p53-null SAOS-2 cells (Figure S7A), suggesting that IRTKS could be decrease by DNA damage induced p53 activation. This finding promoted us to assess the dynamics of IRTKS expression. Interestingly, MDM2 overexpression reduced the protein levels of IRTKS (Figure 7B), suggesting that MDM2 may mediate IRTKS degradation. Both in vitro and in vivo ubiquitination assays demonstrated that MDM2 mediated IRTKS ubiquitination in a dose-dependent manner (Figure 7C and 7D). These results strongly indicated that MDM2 as an E3 ubiquitin ligase affected IRTKS stability at high activity levels via ubiquitination and proteasomal degradation.

We then investigated the effect of DNA damage on the interaction of IRTKS, p53 and MDM2. UV irradiation in HT1080 cells significantly attenuated the interaction between endogenous IRTKS and p53 (Figure S7B). Moreover, the DNA-damaging agent etoposide abolished the IRTKS-p53 interaction and stabilized the IRTKS-MDM2 interaction in U2OS cells when all three proteins were stabilized by MG132 (Figure 7E). These data suggested that DNA damages affected the IRTKS-p53 and IRTKS-MDM2 interactions differently and specifically.

## Discussion

P53-induced transcriptional alteration causes a variety of cell fate changes, including apoptosis, growth arrest, senescence, DNA repair. For maintenance the homeostasis of cell, various processes are equipped to modulate p53 activity. Adjusting the protein levels and transcriptional activity of p53 are two essential processes for cells to maintain the physiological state [25]. Although polyubiquitination of p53 targets its proteasomal degradation, several studies have demonstrated that p53 ubiquitination also affects its activation. WWP1-mediated p53 ubiqitination causes stabilization of p53 but inactivates p53 transactivation [26]. Ubc13 elicits K63-dependent ubiquitination of p53, increases p53 localization to the cytoplasm and p53 stability, and decreases its transcriptional activity [27]. CUL7 causes only mono- or di-ubiquitination of p53, and promotes cell growth through antagonizing the function of p53 [28]. p53 ubiquitination could be important for keeping p53 inactive. De-ubiquitination of p53 is essential for p53-mediated mitochondrial apoptosis and for recycling of p53 from cytoplasm to nucleus [21], [29]. In this study, our data showed that IRTKS decreased p53 activity and increased low levels of MDM2-mediated p53 ubiquitination via binding to both p53 and MDM2.

How, then, IRTKS promoted MDM2-mediated p53 ubiquitination but not degradation via direct interaction? N-terminal transactivation domain of p53 has been characterized as the primary binding site to MDM2 [23], [30], [31]. This primary interaction has been documented to be essential, but not sufficient, to target p53 degradation. The second binding site of p53 to MDM2 has been identified in its DNA-binding domain [32], [33]. A conformational change of MDM2 caused by the primary interaction enables binding of the central region in MDM2 to the DNA-binding domain of p53 [34]. The secondary interaction is required for efficient p53 polyubiquitination and proteasomal degradation [35]. p53 mutants which contain single point mutations in p53 DNA binding domain have increased levels of MDM2-dependent ubiquitination, but not degradation of p53 in vivo [36]. Similarly, IRTKS slightly stabilized p53 protein while increased p53 ubiquitination (Figure 3B), implying that the binding of IRTKS and p53 affected the secondary interaction of p53 and MDM2.

Several partners of MDM2, including ARF, L5, L11, and L23, have been reported to bind to the central region of MDM2 [37]–[41]. Most of them inhibit MDM2-mediated p53 ubiquitination and degradation, probably by blocking the secondary interaction between MDM2 and p53. Our data suggest that IRTKS bound to both the central domain of MDM2 and the DNA binding domain of p53, and recruited MDM2 to p53 in unstressed cells. IRTKS stabilized the p53-MDM2 interaction at low levels of MDM2, but it could also block the secondary interaction sites of p53-MDM2 interaction. Therefore, IRTKS enhanced p53 ubiquitination but not degradation in unstressed cells.

The effects of MDM2-mediated ubiquitination on p53 are in a delicate balance to determine the cell fate during normal homeostasis and stress response. The negative feedback loop between MDM2 and p53 ensures that p53 is maintained at low levels under normal conditions or after stress resolved [4]. Interestingly, IRTKS could be also involved in the negative feedback regulation. Here we showed that p53 dissociated with IRTKS after DNA damage. IRTKS polyubiquitination and degradation by high levels of MDM2 might increase the direct interaction of the secondary sites of p53 and MDM2 for p53 polyubiquitination and degradation.

Together, these data led us to propose a novel mechanism for maintaining the balance of MDM2-mediated ubiquitination on p53 during normal homeostasis and stress response. In unstressed cells with low levels of MDM2, IRTKS stabilized the MDM2-p53 interaction and enhanced MDM2-mediated p53 ubiquitination, thereby inhibiting p53 transcriptional activity. In response to DNA damage, the stabilized MDM2-IRTKS dissociated from p53. MDM2 may possess E3 ligase for self-degradation and IRTKS ubiquitination. Late after DNA damage, MDM2, as a p53 target, was up-regulated and high levels of MDM2 mediated the polyubiquitination and degradation of both IRTKS and p53.

Several proteins have been proved to act as a regulator and substrate of MDM2 in the negative feedback regulation loop between p53 and MDM2. MDM2 can downregulate p53 protein synthesis by inducing the polyubiquitination of L26, a ribosomal protein which stimulates translation of p53 mRNA after DNA damage [42]. Another ribosomal protein S7 interacts with MDM2, inhibiting the E3 ligase activity of MDM2 and stabilizing both MDM2 and p53. S7 itself is ubiqutinated by MDM2 and ubiquitinated S7 selectively destabilizes MDM2, enhancing the p53 stress response [43]. Herein, we demonstrated IRTKS can regulate p53 by modulating the p53-MDM2 interaction as a scaffold protein and substrate of MDM2.

Previous studies suggest that IRSp53-like proteins, including IRTKS, contribute to actin dynamics and membrane protrusion. This function is mainly achieved by the IMD which is the most conserved domain of these proteins [12]. The IMD, also known as an I-BAR (inverse Bin-amphiphysin-Rvs167) domain, binds F-actin, RAC, and lipid, triggering protrusive membrane deformation [10]. Here we show that the IMD of IRTKS was responsible for its association with p53 and MDM2, indicating the IMD-contain proteins might have combination functions of cell mobility and apoptosis. This finding suggests that the IRSp53-like proteins may have diverse functions in cytoplasm and nucleus. While the middle region of IRSp53 is a partial CRIB domain which binds to active CDC42, IRTKS can not interact with CDC42 and its middle region provided a second binding site for IRTKS-p53 interaction. Therefore, it remains to determine whether the other IRSp53-like proteins can affect p53 ubiquitination.

As a tumor suppressor, p53 is presumed as a protein that acts negatively to prevent motility and tumor metastasis [44]. Actin polymerization and membrane deformation are important processes for cell mobility [45]. Recently, a p53 cofactor JMY, has been identified as an actin nucleation factor which is involved in to cell motility [46]. Under DNA damage, JMY augments in nucleus and drives the p53 transcription, its influence on cell motility is reduced [47]. As IRTKS was involved in both actin dynamic and p53 modification, it remains to investigate whether IRTKS is another protein for coordinating cell mobility with the p53 response. And further studies need to address the behavior of IRTKS in tumor as it has diverse functions in inhibiting p53 activation in nucleus and promoting cell mobility in cytoplasm.

## Materials and Methods

### Plasmids and antibodies

The entire open reading frame of human IRTKS was amplified from a human fetal liver cDNA library (Clontech) and subcloned into mammalian cell expression vectors pFLAG-CMV 2 (Sigma) for mammalian expression. Plasmids for expressing p53 and MDM2 in mammalian cells were constructed in pcDNA 3.0. For in vitro experiment, IRTKS, p53 and MDM2 including their truncations were constructed by PCR, and subcloned into pGEX-5X-1 and PET-28a. For immunoblotting and immunoprecipitation detection of endogenous IRTKS, a rabbit polyclonal antibody was raised against GST-IRTKS. p53 antibody DO-1 and FL393, MDM2 antibody H-221 (Santa Cruz) were used for immunoprecipitation. p53 antibody 1C12 (Cell Signaling Technology) and MDM2 antibody H-221 were used for immunofluorescence staining. p53 antibody DO-1 and FL393, MDM2 antibody H-221, cleaved PARP (Asp214) antibody (Cell Signaling Technology ) and PIG3 antibody A-5 (Santa Cruz) were used for Western blotting.

### Cell culture

Human HT1080, U2OS, SAOS-2, H1299 and HEK 293 cells (ATCC) were cultured at 37°C in DMEM supplemented with 10% fetal bovine serum (FBS). p53–Mdm2 double knockout mouse embryonic fibroblast cell line was kindly provided by Prof. Long Yu (Fudan University). Cells were transfected using FuGENE HD or FuGENE 6 by the manufacturer's protocol (Roche). Short interfering RNA (siRNA) oligonucleotides were transfected into cells using Lipofectamine 2000 by the manufacturer's instructions (Invitrogen). The IRTKS siRNA (siIRTKS) sequence was 5′-GCUUAAGCAAAUCAUGCUU-3′. The same sequence with two nucleotides changed (5′-GCUUGAGCAAAUCAUACUU-3′) was used as a specific RNAi control(siIRTKSm). The p53 siRNA sequence was 5′-GACUCCAGUGGUAAUCUAC-3′ and MDM2 siRNA sequence was 5′-CCACCUCACAGAUUCCAGC-3′. Luciferase siRNA (5′-CUUACGCUGAGUACUUCG ATT-3′) was used as a control siRNA(siLUC).

### Apoptosis assay

HT1080 cells transfected with FLAG-IRTKS or IRTKS siRNA were UV-irradiated (60 J/M^2^). The cell apoptosis was detected by using Annexin V-FITC apoptosis detection kit (Pharmingen) according to the manufacturer's instruction. Cells that stain positive for Annexin V-FITC and negative for propidium iodide were counted as apoptotic cells.

### Immunofluorescence microscopy

Cells cultured on coverslips were washed twice with cold PBS. Cells were fixed with 4% paraformaldehyde for 10 min, permeabilized with 0.1% Triton X-100 for 10 min, blocked with 5% bovine serum albumin (BSA) and incubated with anti-IRTKS and anti-p53 or anti-MDM2 antibodies as indicated, followed by incubation with Alexa-546 conjugated anti-rabbit IgG and Alexa-488 conjugated anti-mouse IgG antibody (Invitrogen). The cells were mounted with DAPI-containing medium (Sigma) and the images were acquired with a confocal microscope.

### Immunoprecipitation and Western blotting

Whole cell lysates were prepared in the lysis buffer (50 mM Tris at pH 8.0, 150 mM NaCl, 1% Triton X-100, 1× complete protease cocktail, 5 mM sodium vanadate, and 10% glycerol). The lysates were then immunoprecipitated with the indicated antibodies and isotype-matched control antibodies plus protein G Sepharose (Roche) for at least 4 h or overnight. Beads were washed three times with the lysis buffer, and boiled in 2× loading buffer. Protein samples were resolved by SDS–PAGE and transferred onto nitrocellulose membrane, which was blocked in 5% skim milk in PBS-Tween and probed with the indicated antibodies. The membrane was scanned by Odyssey infrared imaging system (LI-COR) at a 700–800 channel wavelength.

### Subcellular fractionation

Nuclear and cytoplasmic fractions were prepared using NE-PER® Nuclear and Cytoplasmic Extraction Reagents (Pierce biotechnology) according to the manufacturers' protocol. Purity of fractions was determined by western blotting with anti-HDAC1 and anti-Tublin (Santa Cruz Biotechnology) antibodies.

### In vivo ubiquitination and neddylation assay

Cells were transfected with His-ubiquitin, HA-ubiquitin or HA-NEDD8 and other indicated plasmids. Twenty hours after transfection, cells were treated with 25 µM MG132 for 4–6 h and scraped into PBS and pelleted by centrifugation. For transfections with His-ubiquitination, cells were resuspended in 1 ml of denaturing buffer (6 M guanidinium-HCl, 0.5% Triton, 10 mM imidazole, 20 mM Tris-HCl [pH 8], 0.5 mM DTT, and 0.5 mM iodoacetamide) with mild sonication. His–Ub conjugated proteins were purified by nickel chromatography (Ni-NTA Agarose; Qiagen). Nickel bound ubiquitinated proteins were subjected to Western blotting analysis using anti-p53 Ab (DO-1). For transfections with HA-ubiquitin or HA-NEDD8, cells were resuspended in 1% SDS in TBS and boiled twice for 5 min each time. The lysated were diluted to a solution with final concentrations of 0.2% SDS and 1% Triton X-100, and immunoprecipitated with anti-HA antibody (Sigma). The ubiquitinated or neddylated proteins were analyzed with Western blotting by using anti-p53 Ab (DO-1).

### In vitro ubiquitination assay

Purified recombinant proteins as indicated were incubated in 20 µl of reaction buffer (50 mM Tris-HCl pH 7.6, 1 mM DTT, 2 mM ATP and 5 mM MgCl_2_), in the presence of 50 ng of E1 and 100 ng E2 (UbcH5a) enzymes and 10 µg of ubiquitin (Boston Biochem, Boston, MS). After incubation at 32°C for 1 h, the reaction products were analyzed by SDS-PAGE followed by Western blotting.

## Supporting Information

Figure S1 **IRTKS inhibited apoptosis.** (A) Overexpression of IRTKS inhibited UV-induced apoptosis. HT1080 cells were transfected with pCMV-FLAG-IRTKS for overexpression of IRTKS, UV-irradiated (60 J/M^2^) and analysed by FACS with Annexin V FITC/PI staining. For evaluation of cells undergoing apoptosis, cells were stained by both Annexin V-FITC and PI, and the PI-positive cells were excluded from the measurement. (B) Knockdown of IRTKS enhanced UV-induced apoptosis in HT1080 cells. HT1080 cells were transfected with IRTKS siRNA and control siRNA, UV-irradiated and analysed by FACS with Annexin V FITC/PI staining. (C) HT1080 cells were transfected with IRTKS siRNA and control siRNA, UV-irradiated and analysed with anti-cleaved PARP, anti-p53 and anti-IRTKS by Western blotting at indicated time points. Note that the cleaved PARP was increased earlier in the cells with IRTKS knockdown and IRTKS was decreased after UV irradiation. (D) Overexpression of IRTKS reduced the apoptosis induced by p53. SAOS-2 cells were examined by phase contrast microscopy after infection with Ad-p53, Ad-IRTKS and control virus: AdGFP, for 48 h. (E) Knockdown of IRTKS increased the apoptosis induced by p53.
(TIF)

Figure S2 **IRTKS interacted with p53.** (A) HT1080 cells were pretreated with the proteasome inhibitor MG132. The endogenous interaction of IRTKS and p53 was analysed by immunoprecipitation assay with anti-p53 antibody. (B) The fine-mapping of the interaction sites of p53 binding to IRTKS by GST-pulldown assays.
(TIF)

Figure S3 **IRTKS could not affect the phosphorylation and acetylation of certain amino acid residues of p53.** (A) The effect of IRTKS overexpression on p53 phosphorylation. HT1080 cells transfected with IRTKS or control plasmid were untreated, UV-irradiated (60 J/M^2^) or treated with doxorubicin (DOX, 1 µM) for 20 h and collected in lysis buffer. The cell lysates were immunoprecipitated (IP) with anti-p53 antibodies and analyzed by Western blotting using anti-phospho-p53 (S15, S37, S46) and anti-p53 antibodies. (B) The effect of IRTKS depletion on p53 phosphorylation. HT1080 cells transfected with IRTKS siRNA were treated with UV irradiation and harvested at indicated time. p53 phosphorylation were analyzed by Western blotting using anti-phospho-p53 (S15, S20 and S392). (C) and (D) HT1080 cells with overexpression (C) or knockdown (D) of IRTKS were UV-irradiated for 20 hr. The cells treated with trichostatin A (10 nM) for 4 hr and collected in lysis buffer. The cell lysates were immunoprecipitated (IP) with anti-p53 antibodies and analyzed by Western blotting using anti-acetyl-p53 (K382) and anti-p53 antibodies.
(TIF)

Figure S4 **IRTKS enhanced p53 ubiquitination.** (A) IRTKS enhanced ubiquitination of endogenous p53. HT1080 cells transfected with IRTKS or control plasmid were untreated, UV-irradiated (60 J/M^2^) or treated with doxorubicin (DOX, 1 µM) for 20 h. The cells were boiled in denaturing lysis buffer (50 mM Tris pH 7.4, 150 mM NaCl, 1% SDS). The denaturing lysates were diluted with dilution buffer (50 mM Tris pH 7.4, 150 mM NaCl, 1% Triton X-100) until the concentration of SDS reached 0.2% and immunoprecipitated (IP) with anti-p53 antibodies. The immunoprecipitates were analyzed by Western blotting using anti-ubiquitin and anti-p53 antibodies. (B) Knockdown of IRTKS inhibited p53 ubiquitination. HT1080 cells transfected with IRTKS siRNA were treated with UV or DOX. The cell lysates were immunoprecipitated with anti-p53 antibody. p53 ubiquitination was analyzed by Western blotting with anti-ubiquitin antibody.
(TIF)

Figure S5 **p53–Mdm2 double knockout mouse embryonic fibroblasts were transfected with indicated plasmids.** The cell lysates were loaded on nickel (Ni+)-NTA columns. p53 ubiquitination was analyzed with Western blotting by using anti-p53 antibody (DO-1). Note that IRTKS cloud not increase p53 ubiquitination without MDM2 expression.
(TIF)

Figure S6 **IRTKS promoted MDM2-mediated p53 ubiquitination.** (A) MDM2 induced both mono- and polyubiquitination of p53 in a dose-dependent manner *in vitro*. Western blotting analysis with p53-specific monoclonal antibody (DO-1) of p53 (100 ng) incubated with varying amounts of His-MDM2. (B) In vitro ubiquitination experiments show that IRTKS promoted p53 ubiquitination mediated by MDM2.
(TIF)

Figure S7 **MDM2 mediated IRTKS degradation.** (A) DNA damage could not alter the protein levels of IRTKS in SAOS-2 cell. (B) DNA damage disrupted the IRTKS-p53 interaction. HT1080 cells were exposed to UV radiation (60 J/M^2^) and lysed at the indicate time. The cell lysates were immunoprecipitated with p53 antibody. The association of IRTKS and p53 was detected by Western blotting.
(TIF)
