# Supplementary figures and images for: Insulin Receptor Tyrosine Kinase Substrate Enhances Low Levels of MDM2-Mediated p53 Ubiquitination
Source: PLoS One. 2011 Aug 24;6(8):e23571. doi: 10.1371/journal.pone.0023571 (PMC3160901; doi:10.1371/journal.pone.0023571)

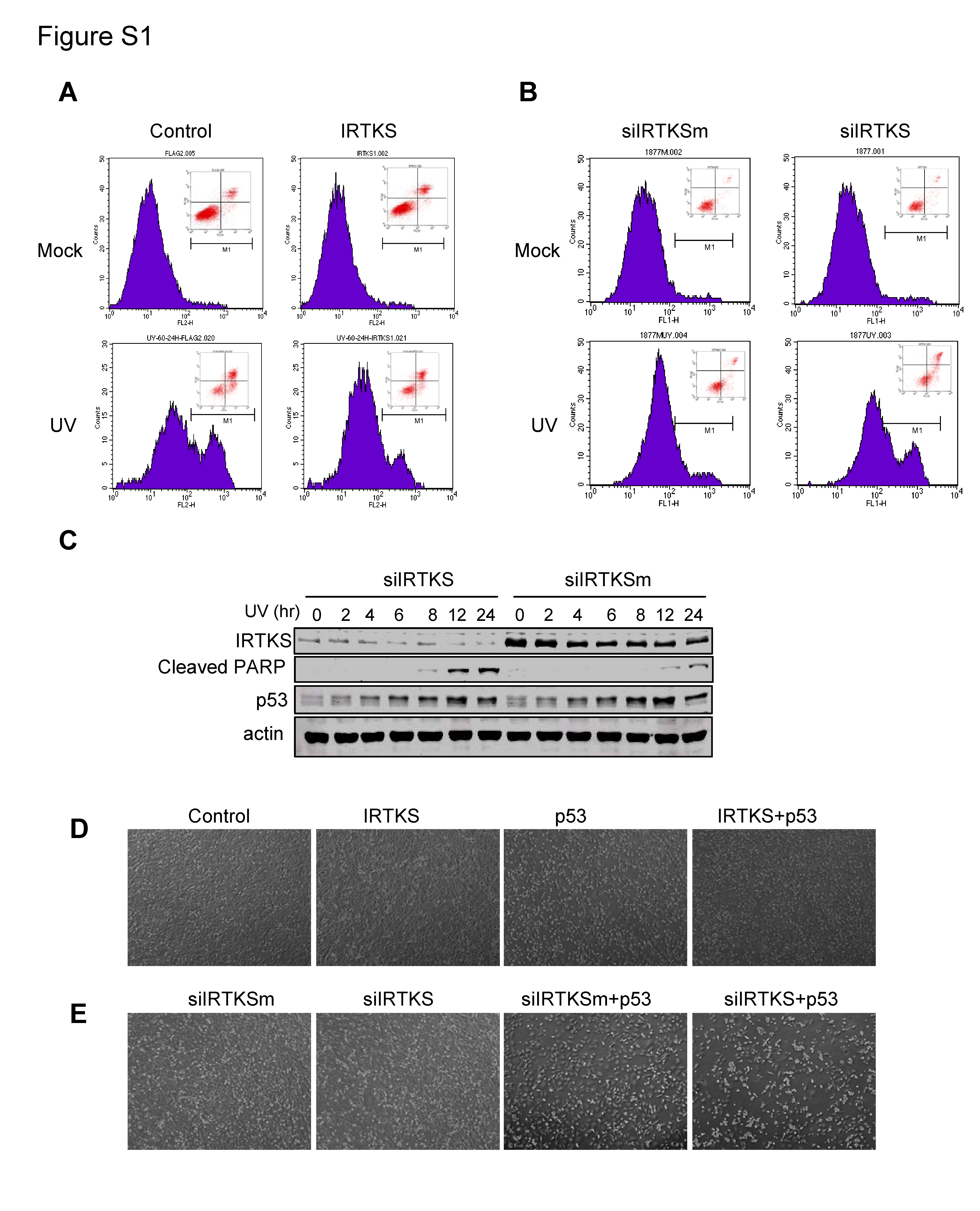

Supplement: Figure S1 — IRTKS inhibited apoptosis. (A) Overexpression of IRTKS inhibited UV-induced apoptosis. HT1080 cells were transfected with pCMV-FLAG-IRTKS for overexpression of IRTKS, UV-irradiated (60 J/M2) and analysed by FACS with Annexin V FITC/PI staining. For evaluation of cells undergoing apoptosis, cells were stained by both Annexin V-FITC and PI, and the PI-positive cells were excluded from the measurement. (B) Knockdown of IRTKS enhanced UV-induced apoptosis in HT1080 cells. HT1080 cells were transfected with IRTKS siRNA and control siRNA, UV-irradiated and analysed by FACS with Annexin V FITC/PI staining. (C) HT1080 cells were transfected with IRTKS siRNA and control siRNA, UV-irradiated and analysed with anti-cleaved PARP, anti-p53 and anti-IRTKS by Western blotting at indicated time points. Note that the cleaved PARP was increased earlier in the cells with IRTKS knockdown and IRTKS was decreased after UV irradiation. (D) Overexpression of IRTKS reduced the apoptosis induced by p53. SAOS-2 cells were examined by phase contrast microscopy after infection with Ad-p53, Ad-IRTKS and control virus: AdGFP, for 48 h. (E) Knockdown of IRTKS increased the apoptosis induced by p53. (TIF) [file pone.0023571.s001.tif]

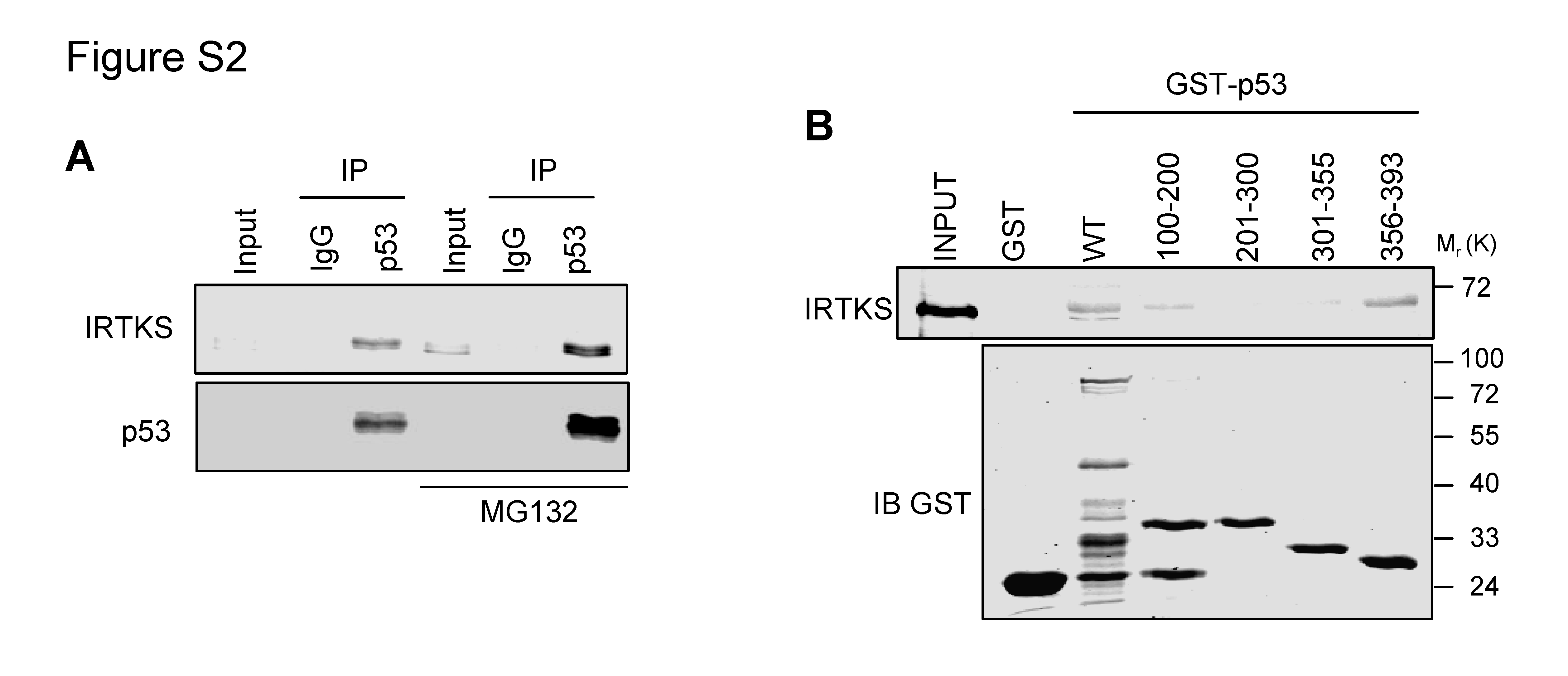

Supplement: Figure S2 — IRTKS interacted with p53. (A) HT1080 cells were pretreated with the proteasome inhibitor MG132. The endogenous interaction of IRTKS and p53 was analysed by immunoprecipitation assay with anti-p53 antibody. (B) The fine-mapping of the interaction sites of p53 binding to IRTKS by GST-pulldown assays. (TIF) [file pone.0023571.s002.tif]

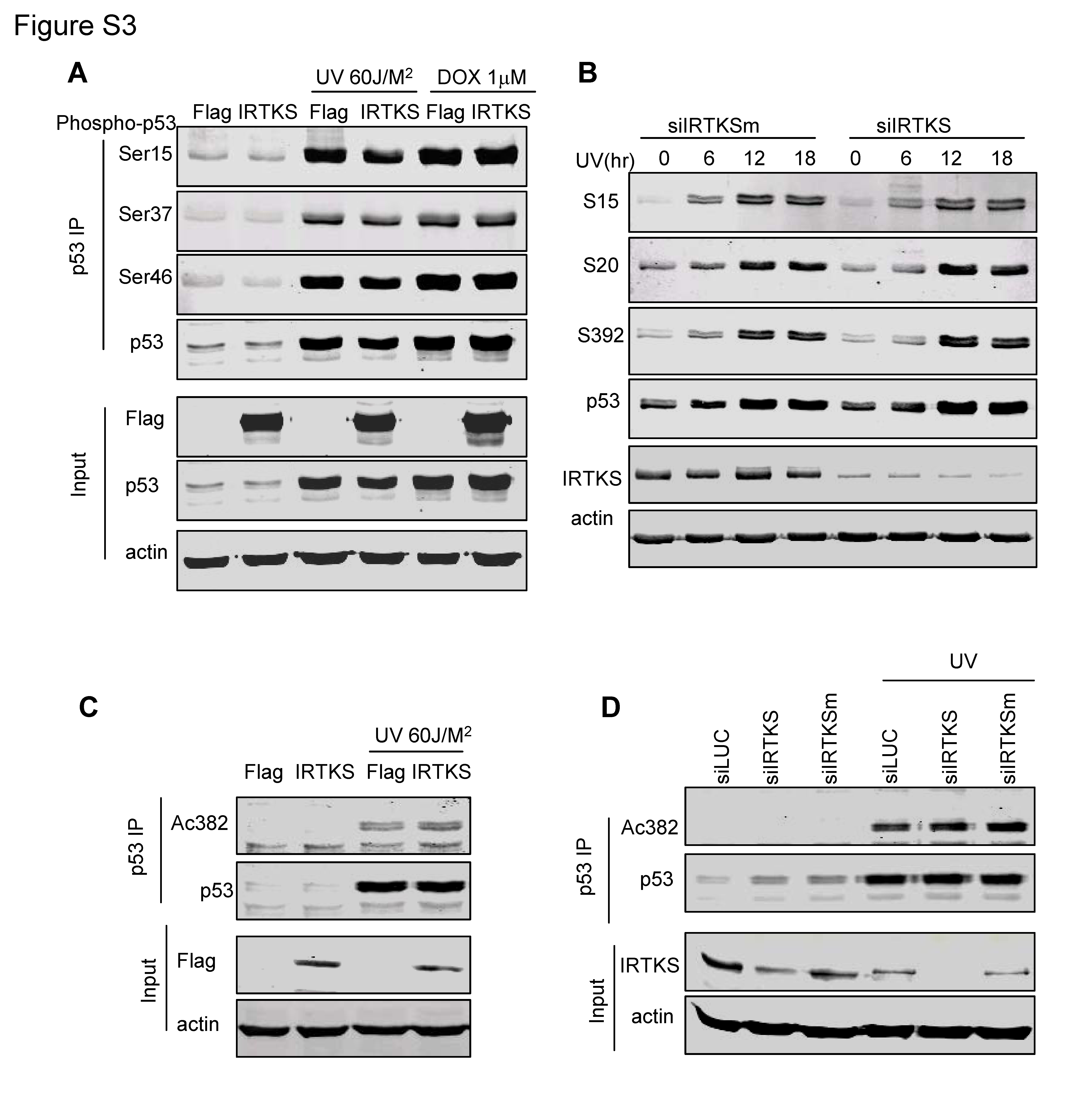

Supplement: Figure S3 — IRTKS could not affect the phosphorylation and acetylation of certain amino acid residues of p53. (A) The effect of IRTKS overexpression on p53 phosphorylation. HT1080 cells transfected with IRTKS or control plasmid were untreated, UV-irradiated (60 J/M2) or treated with doxorubicin (DOX, 1 µM) for 20 h and collected in lysis buffer. The cell lysates were immunoprecipitated (IP) with anti-p53 antibodies and analyzed by Western blotting using anti-phospho-p53 (S15, S37, S46) and anti-p53 antibodies. (B) The effect of IRTKS depletion on p53 phosphorylation. HT1080 cells transfected with IRTKS siRNA were treated with UV irradiation and harvested at indicated time. p53 phosphorylation were analyzed by Western blotting using anti-phospho-p53 (S15, S20 and S392). (C) and (D) HT1080 cells with overexpression (C) or knockdown (D) of IRTKS were UV-irradiated for 20 hr. The cells treated with trichostatin A (10 nM) for 4 hr and collected in lysis buffer. The cell lysates were immunoprecipitated (IP) with anti-p53 antibodies and analyzed by Western blotting using anti-acetyl-p53 (K382) and anti-p53 antibodies. (TIF) [file pone.0023571.s003.tif]

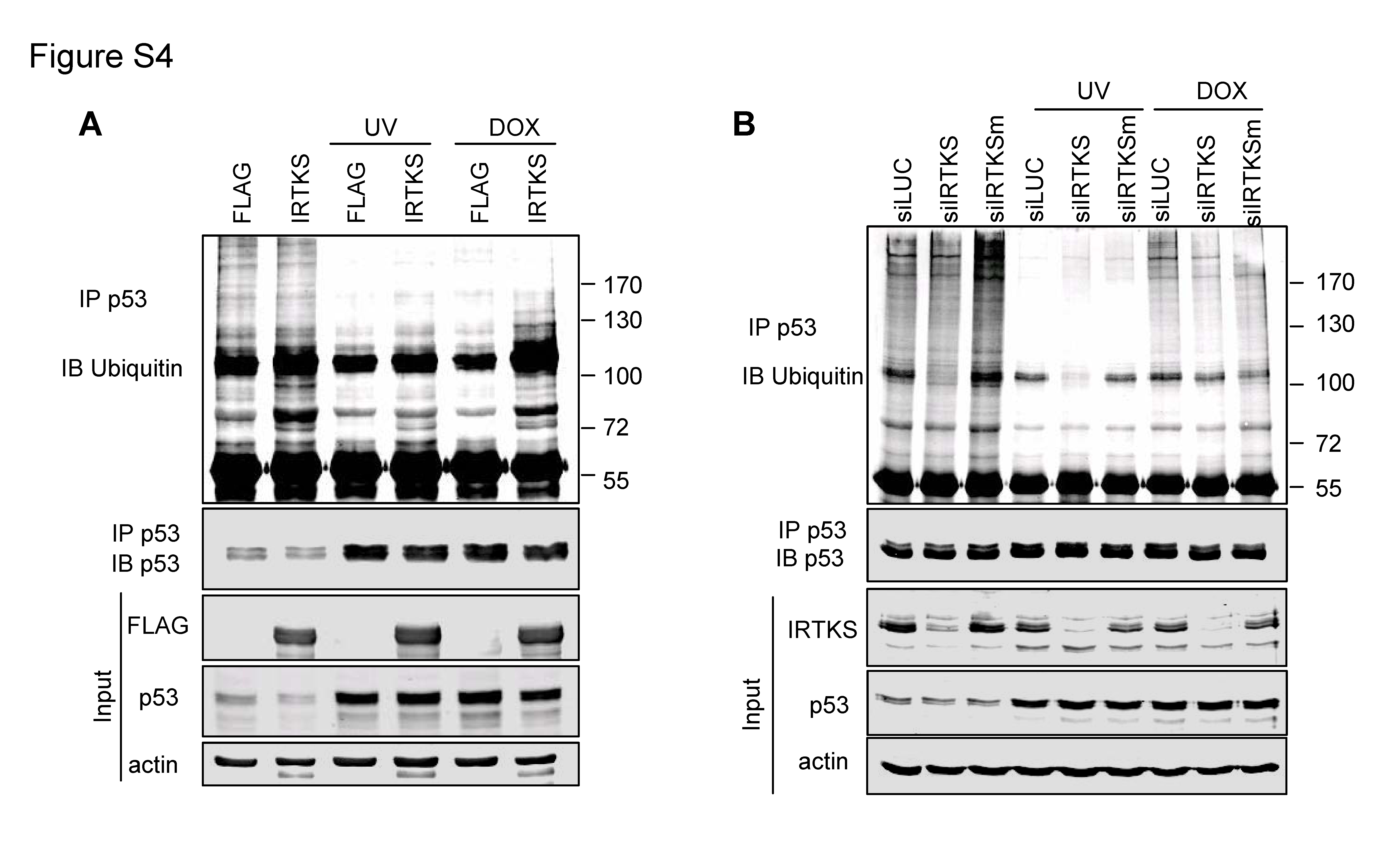

Supplement: Figure S4 — IRTKS enhanced p53 ubiquitination. (A) IRTKS enhanced ubiquitination of endogenous p53. HT1080 cells transfected with IRTKS or control plasmid were untreated, UV-irradiated (60 J/M2) or treated with doxorubicin (DOX, 1 µM) for 20 h. The cells were boiled in denaturing lysis buffer (50 mM Tris pH 7.4, 150 mM NaCl, 1% SDS). The denaturing lysates were diluted with dilution buffer (50 mM Tris pH 7.4, 150 mM NaCl, 1% Triton X-100) until the concentration of SDS reached 0.2% and immunoprecipitated (IP) with anti-p53 antibodies. The immunoprecipitates were analyzed by Western blotting using anti-ubiquitin and anti-p53 antibodies. (B) Knockdown of IRTKS inhibited p53 ubiquitination. HT1080 cells transfected with IRTKS siRNA were treated with UV or DOX. The cell lysates were immunoprecipitated with anti-p53 antibody. p53 ubiquitination was analyzed by Western blotting with anti-ubiquitin antibody. (TIF) [file pone.0023571.s004.tif]

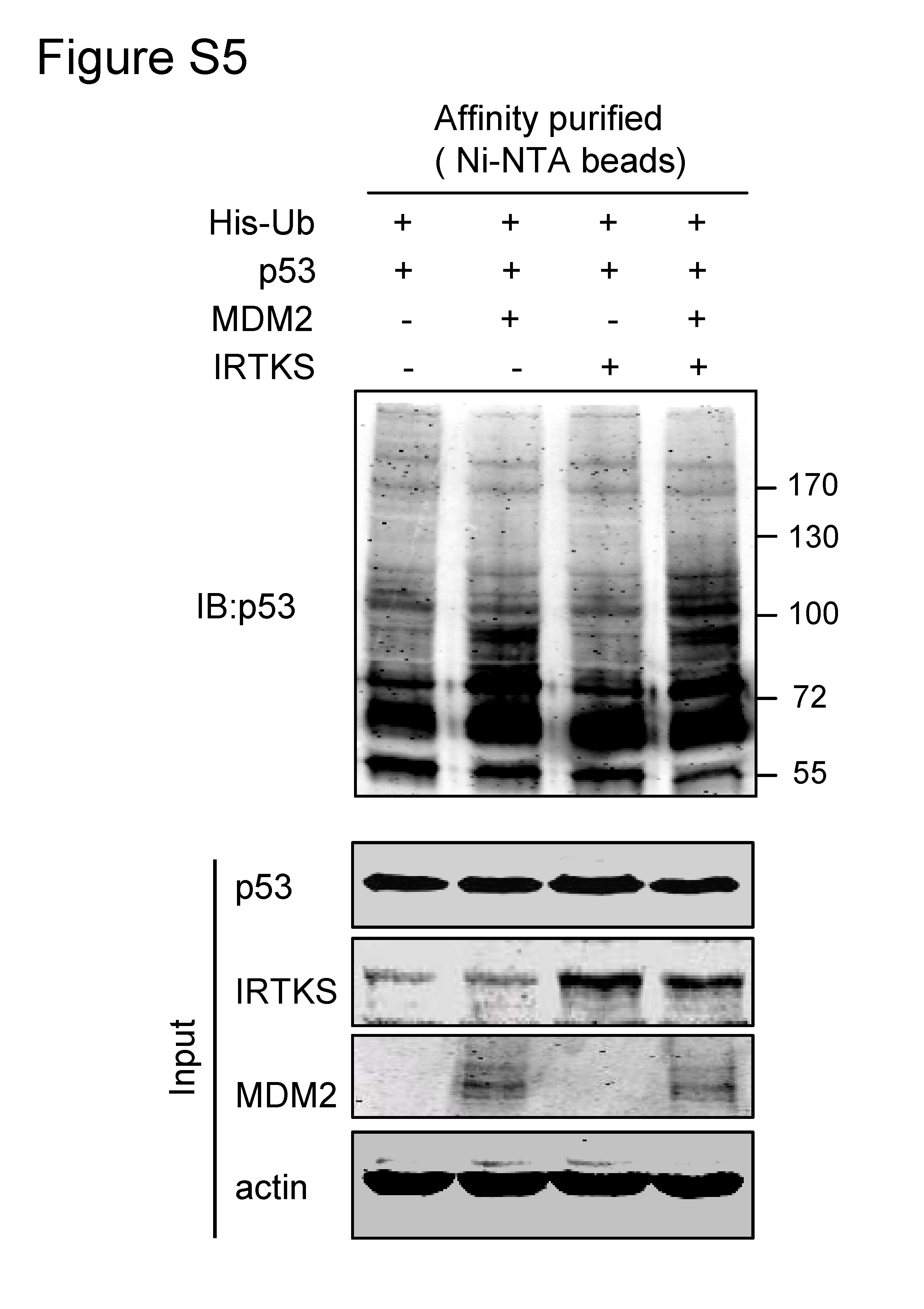

Supplement: Figure S5 — p53–Mdm2 double knockout mouse embryonic fibroblasts were transfected with indicated plasmids. The cell lysates were loaded on nickel (Ni+)-NTA columns. p53 ubiquitination was analyzed with Western blotting by using anti-p53 antibody (DO-1). Note that IRTKS cloud not increase p53 ubiquitination without MDM2 expression. (TIF) [file pone.0023571.s005.tif]

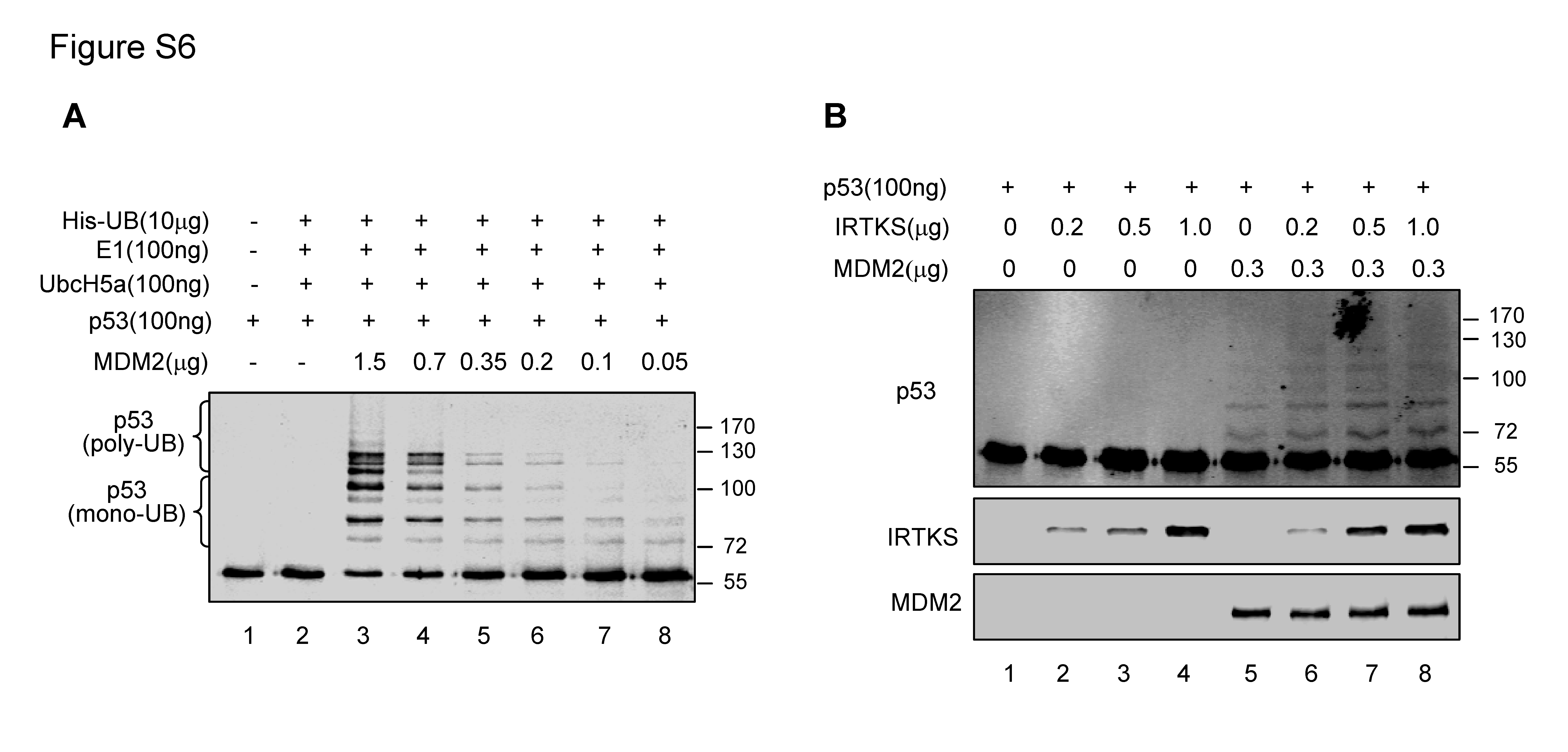

Supplement: Figure S6 — IRTKS promoted MDM2-mediated p53 ubiquitination. (A) MDM2 induced both mono- and polyubiquitination of p53 in a dose-dependent manner in vitro. Western blotting analysis with p53-specific monoclonal antibody (DO-1) of p53 (100 ng) incubated with varying amounts of His-MDM2. (B) In vitro ubiquitination experiments show that IRTKS promoted p53 ubiquitination mediated by MDM2. (TIF) [file pone.0023571.s006.tif]

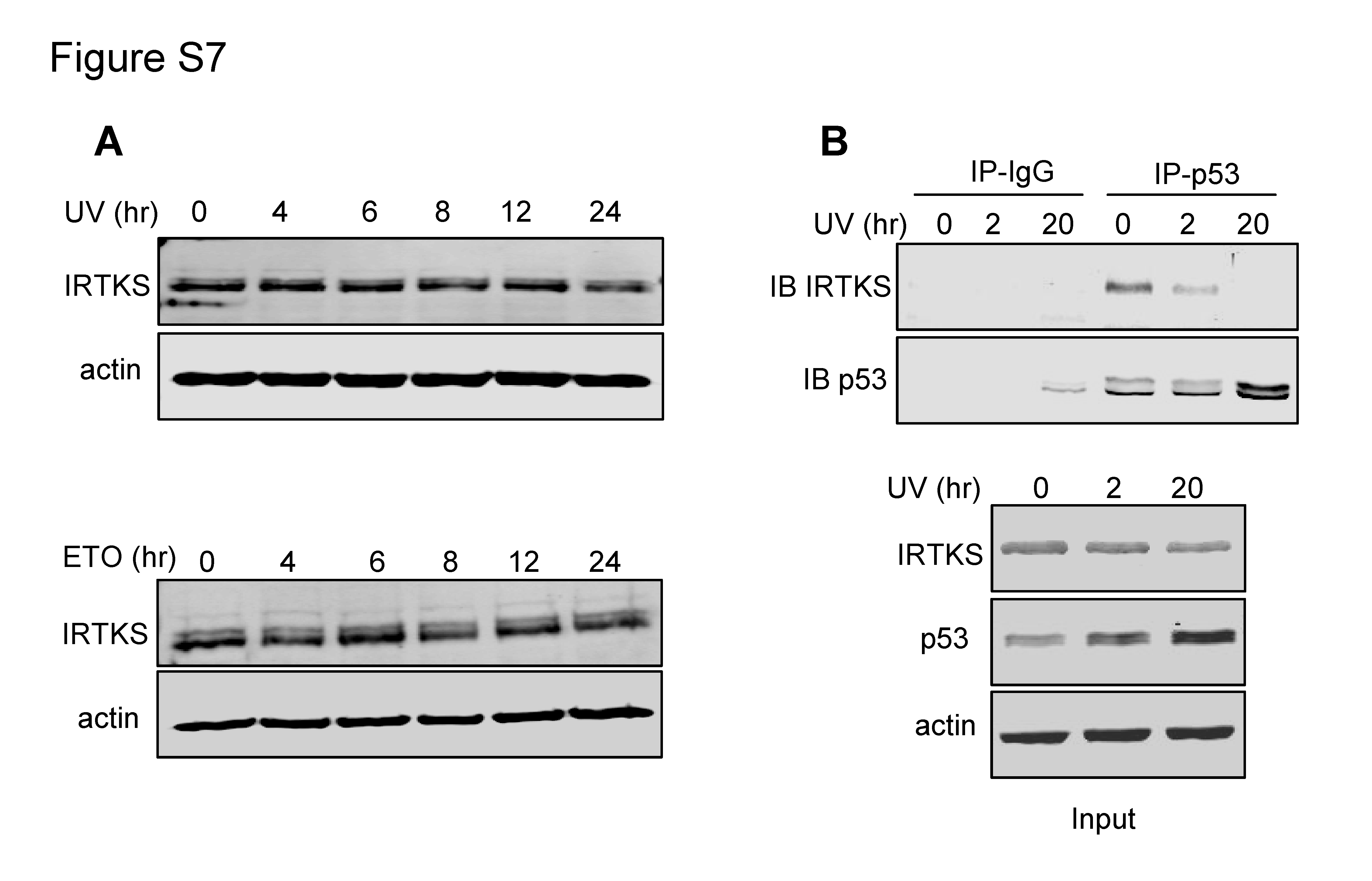

Supplement: Figure S7 — MDM2 mediated IRTKS degradation. (A) DNA damage could not alter the protein levels of IRTKS in SAOS-2 cell. (B) DNA damage disrupted the IRTKS-p53 interaction. HT1080 cells were exposed to UV radiation (60 J/M2) and lysed at the indicate time. The cell lysates were immunoprecipitated with p53 antibody. The association of IRTKS and p53 was detected by Western blotting. (TIF) [file pone.0023571.s007.tif]
